# *EFEMP2* indicates assembly of M0 macrophage and more malignant phenotypes of glioma

**DOI:** 10.18632/aging.103147

**Published:** 2020-05-12

**Authors:** Lijie Huang, Zheng Wang, Yuanhao Chang, Kuanyu Wang, Xun Kang, Ruoyu Huang, Ying Zhang, Jing Chen, Fan Zeng, Fan Wu, Zheng Zhao, Guanzhang Li, Hua Huang, Tao Jiang, Huimin Hu

**Affiliations:** 1 Department of Molecular Neuropathology, Beijing Neurosurgical Institute, Capital Medical University, Beijing, China; 2 Department of Neurosurgery, Beijing Tiantan Hospital, Capital Medical University, Beijing, China; 3 Center of Brain Tumor, Beijing Institute for Brain Disorders, Beijing, China; 4 China National Clinical Research Center for Neurological Diseases, Beijing, China; 5 Chinese Glioma Genome Atlas Network (CGGA), Beijing, China

**Keywords:** *EFEMP2*, malignant glioma, M0 macrophage, immunological responses

## Abstract

Immune response mediated by macrophages is critical in tumor progression and implicates new targets in potential efficient immunotherapies. Tumor associated macrophages (TAM) are divided into either polarized M1 or M2 phenotype depending on different regulators of polarization and pro- or anti-oncogenic roles they play. Glioma-infiltrated TAMs have been newly reported contrary to the current polarization dogma. Instead, macrophages in glioma exhibit a continuum phenotype between the M1- and M2-like TAM that resembling M0 macrophage. Here we proposed an OS (overall survival)-correlated gene *EFEMP2* (*EGF containing fibulin-like extracellular matrix protein 2*) via screening with transcriptional expression levels and methylation data in two glioma databases. *EFEMP2* was found highly expressed in glioma of higher WHO grade and Mesenchymal subtype glioma, and its transcriptional level could predict OS efficiently in validation datasets. EFEMP2 exhibited a remarkable preference of intercellular expression. In vitro assay showed that EFEMP2’s level in medium was closely related to glioma cells’ growth. Moreover, *EFEMP2* expression level was remarkably correlated with immunological responses. M0-like macrophage as a feature of malignancy of glioblastoma revealed distinct assembly in glioma with high level of *EFEMP2*. These results revealed *EFEMP2*’s role as a potential characteristic marker of malignant glioma, which are enriched of M0 macrophage.

## INTRODUCTION

Immunotherapies targeting immune checkpoints showed exciting success in leukemia, melanoma, non-small cell lung cancer and renal carcinoma recent years, and immune checkpoint inhibitor ipilimumab becoming the first checkpoint inhibitor approved for the treatment of cancer [1]. However, although the number of researches and clinical trials on immunotherapies of glioma has increased exponentially in the past few years, most of these efforts failed and almost all glioma are refractory to current immunotherapies [2]. Complexity of the immune microenvironment and the presence of abundant clusters of immune associated cells reduces the efficiency of immunotherapy [3–6]. Glioma is deeply infiltrated with diverse immune cells, including microglia, monocyte-derived macrophages, and myeloid-derived suppressor cells (MDSCs) [7, 8]. These tumor-associated macrophages (TAMs) are domestic microglia or derived from monocytes infiltrated into brain when the blood-brain barrier is compromised during tumor growth and evolve into glioma-associated macrophages [9]. The TAMs fall into either M1 or M2 polarized phenotype depending on environmental context and interrelations with tumor according to the widely accepted polarization dogma. The anti-tumorigenic M1 phenotype is typically acquired after stimulation with Toll-like receptor 4 (TLR4) ligands and IFN-γ, whereas the tumor-supportive M2 phenotype occurs after IL-4, IL-10 or IL-13 exposure [10]. TAMs are M2 polarized in many tumors [11] and it has been reported that macrophages in glioma exhibit a M2-like phenotype. Furthermore, the macrophages in higher grade of glioma are closer to M2 polarity [12–15].

Although the polarization dogma of macrophage is widely used in immunology researches of diverse tumors, the proposition of this mutually exclusive activation theory is based on *in vitro* conditions and meets many difficulties in interpretation of *in vivo* immunological environments of tumor cells [9]. mRNA expression profiles of glioma-associated microglia and macrophages reveal that only partial of the differently expressed genes between TAM in glioma and macrophages in normal brain overlap with reported gene signatures for M1 or M2 (including the three subtypes M2a, M2b and M2c) polarized macrophages. More than half of the differently expressed genes in TAM could not fall into any of the canonical polarization phenotype [16]. Immune phenotyping of glioma-associated macrophages with matched blood monocytes, health donor monocytes, normal brain microglia, nonpolarized M0 macrophages, and polarized M1, M2a, M2c macrophages indicated that macrophages infiltrated in glioma tissue keep a continuum statue between the M1- and M2-like phenotype, and more resemble M0 macrophage phenotype [17]. These researches pointed out that the phenotype of glioma-associated macrophages might be quite different from the other malignant solid tumors and is prone to M0-like phenotype. Although M0-like characteristic of glioma-associated macrophages has been proposed recently, specialized researches of this feature have been seldom reported.

In our previous exploration for the critical factors in malignant progression of glioma, we analyzed mRNA expression and methylation dataset to scan OS-correlated genes through shuttling between the TCGA (The Cancer Genome Atlas) database and the CGGA (Chinese Glioma Genome Atlas) database. *EFEMP2* (*EGF containing fibulin-like extracellular matrix protein 2*) is one of the 7 filtered genes [18]. Now we found that high expression of *EFEMP2* not only remarkably reflected a more malignant phenotype of glioma, but also indicated assembly of M0-like macrophage.

## RESULTS

### *EFEMP2* expression level is correlated with glioma grade and shows a subtype preference

We had performed previous studies to scan for the critically important genes in glioma origin or development [18]. Via investigations of transcriptome and promoter methylation differences between patients of malignant glioma with short (less than one year) and the patients with long (more than three years) survival in CGGA, and validated the differences in TCGA, we had obtained 7 genes that might play critical roles in glioma progression [18]. *EFEMP2* encoding EGF containing fibulin-like extracellular matrix protein 2 was among the 7 filtered genes. Lines of evidence proposed oncogenic feature of *EFEMP2* [19, 20]. Thus, we chose *EFEMP2* for further validation.

**Figure 1:**
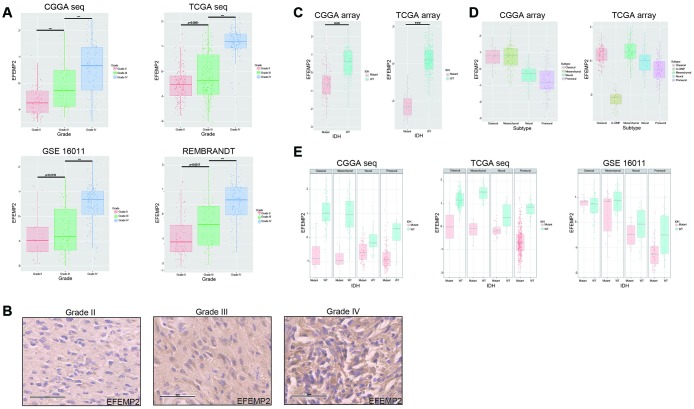
**WHO grade, *IDH1* mutation and transcriptomic subtype preferences of *EFEMP2* expression.** (**A**) The correlation of *EFEMP2* expression level with WHO grade. *EFEMP2* expression levels in glioma of WHO grade II-IV in CGGA RNA-seq, TCGA RNA-seq, GSE16011 and REMBRANDT databases. ***p < 0.0001. (**B**) EFEMP2 expression in glioma specimens determined by IHC analysis. Scale bar, 60 μm. (**C**) The relationship between *EFEMP2* transcription level and *IDH1* mutation in CGGA and TCGA mRNA array datasets. ***p < 0.0001. (**D**) The relationship between *EFEMP2* transcription level and transcriptomic subtype classification in CGGA and TCGA mRNA array datasets. (**E**) Correlation of *EFEMP2* expression and *IDH1* mutation in each transcriptomic subtype in CGGA and TCGA mRNA sequencing data, and GSE16011 dataset.

To ask if *EFEMP2* is involved in malignant progress of glioma, we compared its expression levels in different WHO grades in CGGA mRNA sequencing dataset, TCGA mRNA sequencing dataset of glioma, GSE16011 and REMBRANDT datasets. Except for grade II to grade III in GSE16011 dataset (*p* = 0.2110), *EFEMP2* expression levels increased along with grade progression very significantly (*p* < 0.0001, Figure 1A). We evaluated the expression of EFEMP2 in human glioma specimens and observed that EFEMP2 was indeed highly expressed in GBM specimens (Figure. 1B).

Since it has been widely recognized that *IDH1* mutation is a critical driver of low grade glioma [21], we explored the relationship between *EFEMP2* transcription level and *IDH1* mutation. In both the CGGA (all grades, n = 297) and TCGA (glioblastoma, n = 418) array datasets, patients with strong *EFEMP2* expression were primarily harboring wild type *IDH1*, whereas most of the ones with low *EFEMP2* expression harbored *IDH1* mutation (Figure 1C). The correlation between *EFEMP2* expression and glioma subtypes could also reflect the oncogenic characteristics of *EFEMP2* (Figure 1D). The *EFEMP2* mRNA expression in the four different transcriptional characteristic subtypes were quite different in CGGA (all grades, n = 301). Patients with strong *EFEMP2* expression were mainly concentrated in Classical subtype and Mesenchymal subtype. In TCGA (glioblastoma, n = 520), patients with high expressions of *EFEMP2* were concentrated in Classical and Mesenchymal subtypes, whereas patients with weak *EFEMP2* expressions primarily presented as G-CIMP and Proneural subtypes, which are usually associated with optimistic outcomes [22].

The correlation between *EFEMP2* expression level and *IDH1* mutation statue was further investigated in each molecular subtype based on datasets of CGGA mRNA sequencing, TCGA mRNA sequencing and GSE16011. Except for Classical subtype in GSE16011, glioma with wild type *IDH1* harbored higher *EFEMP2* transcriptional levels in each subtype of these three datasets (Figure 1E).

### EFEMP2 enhances the tumorigenicity of GBM cells in vitro

**Figure 2:**
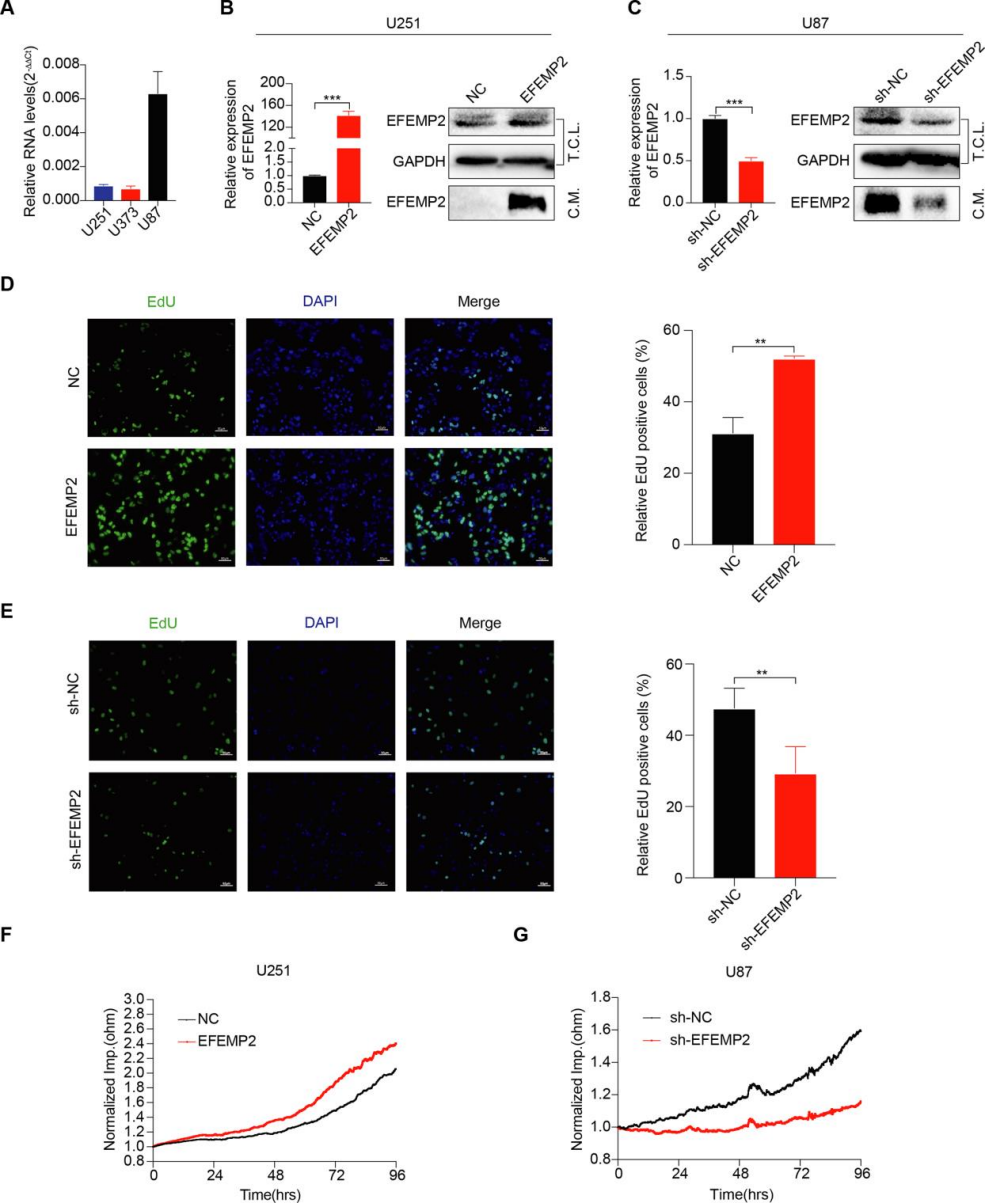
**EFEMP2 promotes GBM cell proliferation.** (**A**) The expression of EFEMP2 was detected in three GBM cell lines by RT-qPCR. GAPDH was used as an internal reference. (**B**) RT-qPCR analysis of EFEMP2 expression in U251 cells overexpressing EFEMP2. Statistical significance was assessed using two-tailed Student’s t test. ***P < 0.001 (left). Western blotting (WB) analysis of EFEMP2 protein level in total cell lysates (T.C.L.) or conditioned media (C.M.) of cells with either vector or EFEMP2 stably overexpressed (right). (**C**) RT-qPCR analysis of EFEMP2 expression in U87 cells knocking down EFEMP2. Statistical significance was assessed using two-tailed Student’s t test. ***P < 0.001 (left). WB analysis of EFEMP2 protein level in total cell lysates (T.C.L.) or conditioned media (C.M.) of cells with either vector or EFEMP2 stably low expressed (right). (**D** and **E**) Proliferation of stable overexpressing (**D**) or knockdown (**E**) EFEMP2 cells as measured by EdU (green) uptake. Quantification of proliferation was measured by % EdU expressing cells / total cell number. Statistical significance was assessed using two-tailed Student’s t test. **P < 0.01. Scale bar, 60 μm. (**F** and **G**) The growth of cells with stable overexpressing (**F**) or knockdown (**G**) EFEMP2 was measured by Electric Cell-substrate Impedance Sensing (ECIS).

To investigate the role of EFEMP2 in GBM tumorigenesis, we stably overexpressed EFEMP2 in U251 cells, which express relatively low levels of EFEMP2 (Figure 2A). RT-qPCR and Western blotting analyses confirmed increased *EFEMP2* RNA and protein expression in U251 cells. According to other studies, EFEMP2 is a secreted protein (Figure 2B). We collected cell culture supernatants and found that the expression of EFEMP2 was more pronounced (Figure 2B). Next, DNA synthesis and cell growth were determined by EdU assays and Electric Cell-substrate Impedance Sensing (ECIS), respectively. The EdU assays revealed that stable overexpression of EFEMP2 significantly increased the DNA synthesis capability of U251 cells (Figure 2D). Otherwise, the growth rate of U251 cells with EFEMP2 overexpression were increased compared with the negative control group (Figure 2F). Next, we selected to knockdown EFEMP2 stably in U87 cells, which exhibit high levels of baseline EFEMP2 (Figure 2A). The successful interference of EFEMP2 in U87 was confirmed by RT-qPCR and Western blotting (Figure 2C). As expected, the DNA synthesis capability and the growth rate of U87 cells with EFEMP2 silencing were decreased compared with the negative control group (sh-NC) (Figure 2E, G). Taken together, these results indicated that EFEMP2 might play a role in maintaining oncogenesis of GBM cell lines in vitro.

### Transcriptional level of *EFEMP2* could effectively predict overall survival and progression-free survival of glioma patients

**Figure 3:**
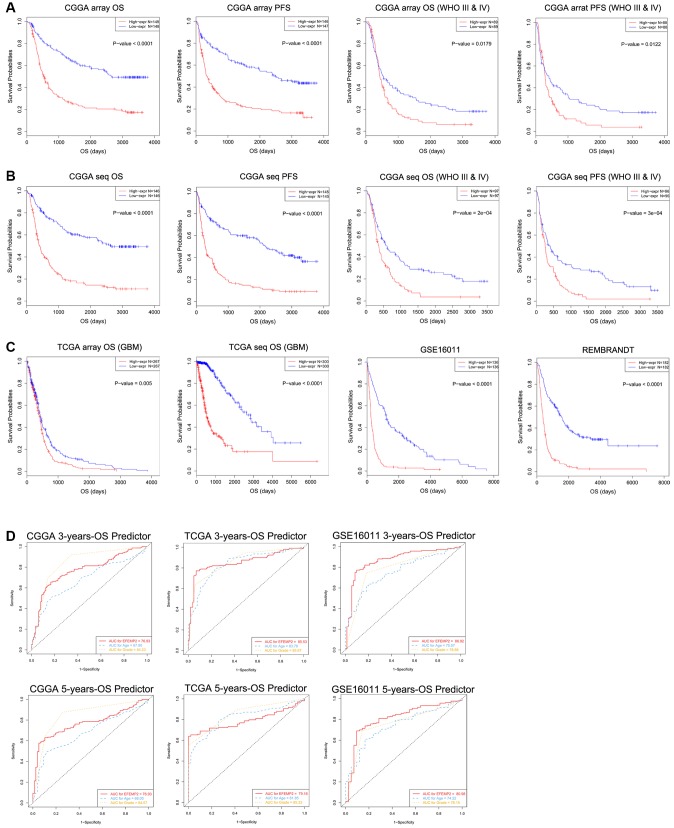
**Patients with higher *EFEMP2* transcription level exhibits poorer OS and PFS.** (**A**) The half of patients with higher *EFEMP2* expression exhibited shorter OS and PFS in Kaplan-Meier analyses based on CGGA mRNA array dataset. (**B**) The half of patients with higher *EFEMP2* expression exhibited shorter OS and PFS in Kaplan-Meier analyses based on CGGA mRNA sequencing dataset. (**C**) Kaplan-Meier analyses of OS based on TCGA mRNA array, TCGA mRNA sequencing data, GSE16011 and REMBRANDT datasets. (**D**) The ROC curves indicating the sensitivity and specifcity of predicting 3- or 5-years of overall survival with *EFEMP2*-level in CGGA, TCGA, or GSE16011 database.

*EFEMP2* expression level was sufficient to predict overall survival (OS) and progression-free survival (PFS) of patients with glioma in four datasets. The patients with both LGG (low-grade glioma) and HGG (high-grade glioma) or only HGG (WHO III and IV grade) were divided into two groups upon their *EFEMP2* expression level derived from CGGA mRNA array (Figure 3A). The half of patients with higher *EFEMP2* expression exhibited shorter OS and PFS in Kaplan-Meier analyses. The differences of the OS and PFS were very significant (*p* < 0.0001) in patients of all grades (including HGG and LGG) and also quite remarkable (*p* = 0.0179 for OS and *p* = 0.0122 for PFS) in patients of HGG. To avoid the bias brought by technical platform, we repeated the OS and PFS analyses with *EFEMP2* expression level derived from CGGA mRNA sequencing dataset (Figure 3B). The half of patients with higher *EFEMP2* expression exhibited shorter OS and PFS in either all grades or HGG glioma. And the OS/PFS distinguishing effects of *EFEMP2* expression level were all very significant (*p* < 0.0001). Kaplan-Meier analyses based on TCGA mRNA array or sequencing data of glioma, GSE16011 and REMBRANDT confirmed the OS distinguishing effects of *EFEMP2* expression levels (Figure 3C).

The specificity and sensitivity of *EFEMP2* mRNA-level in indications of 5 or 3 years of survival were tested in CGGA, TCGA mRNA sequencing data, and GSE16011 mRNA array data via ROC tests (Figure 3D). The area under curves (AUCs) for *EFEMP2* transcriptional level in prediction of 5 and 3 years of overall survival in CGGA data were larger than those of “age”, despite smaller than the AUCs of “grade”. In TCGA data, the AUC for 3-years-OS was larger than that of “age” but smaller than “grade”, whereas the AUC for 5-years-OS was smaller than both “age” and “grade”. In GSE16011 data, the AUCs for *EFEMP2* transcriptional level in prediction of 5 and 3 years of overall survival were both larger than “age” and “grade”.

**Figure 4:**
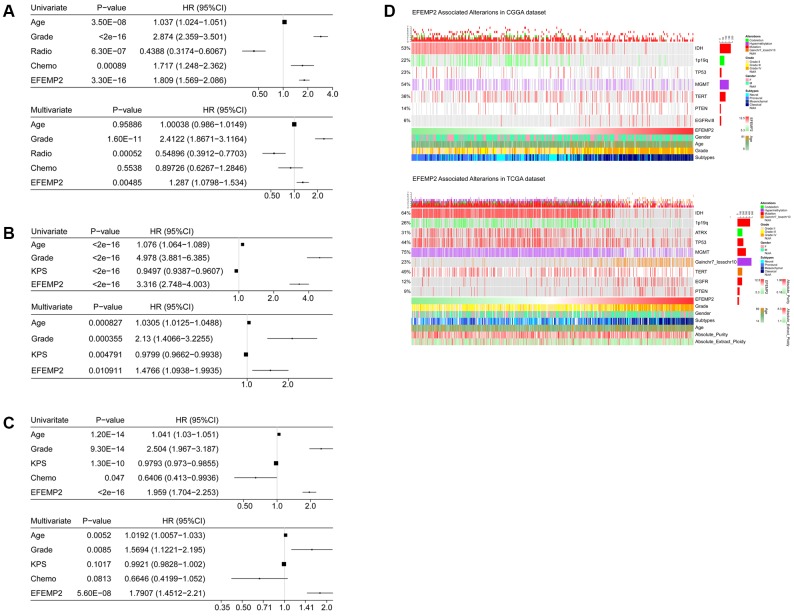
**Univariate and Multivariate Cox regression analyses and correlations with classic genetic alterations of *EFEMP2.***(**A**–**C**) Univariate and Multivariate Cox regression analyses of *EFEMP2* expression level and several other clinical variables in CGGA and TCGA mRNA sequencing data, and GSE16011 data. (**D**) Correlations of *EFEMP2* with classic genetic alterations of glioma. Grey background indicates wild-type or intact genes or chromosomes.

Univariate and Multivariate Cox regression analyses were used to reveal the prognostic efficacy of *EFEMP2*. In all three datasets, including CGGA, TCGA and GSE16011 dataset, *EFEMP2* expression levels exhibited independent prognostic value for glioma patients (Figure 4A–4C).

### Characterization of the correlations of *EFEMP2* with classical genetic alterations of glioma

We investigated the correlation between *EFEMP2* expression level and classical genetic alterations in CGGA and TCGA dataset with Oncoprint plots, generated with *ComplexHeatmap* package developed by Gu, Z. et al. [23] In CGGA dataset, we noticed that, with increasing expression of *EFEMP2*, patients intended to harbor wild type *IDH*, intact 1p19q, *PTEN* mutation and EGFRvIII mutationin their tumors, whereas these classical genetic alterations were known to be indicators of malignant phenotypes and poor outcomes (wild-type *IDH* [21, 24], intact 1p19q [25], *PTEN* mutation [26] and EGFRvIII mutation [27]). In TCGA dataset, we observed even more robust results due to larger sample size. Furthermore, less *ATRX* mutations [28] and *TP53* mutations [29] were observed in high-*EFEMP2*-expressed samples, while significant gain of chromosome 7 and loss of chromosome 10 [30] occurred more frequently in these samples, suggesting malignant properties of these samples (Figure 4D). The coincidences of high-*EFEMP2-*expression with the classical genetic alterations indicating malignancy, and the exclusivity of high-*EFEMP2-*expression with the indicator of positive prognosis descripted the oncogenic nature of *EFEMP2* which is closely correlated to malignant phenotypes of glioma.

### *EFEMP2* indicates assembly of M0 macrophage

**Figure 5:**
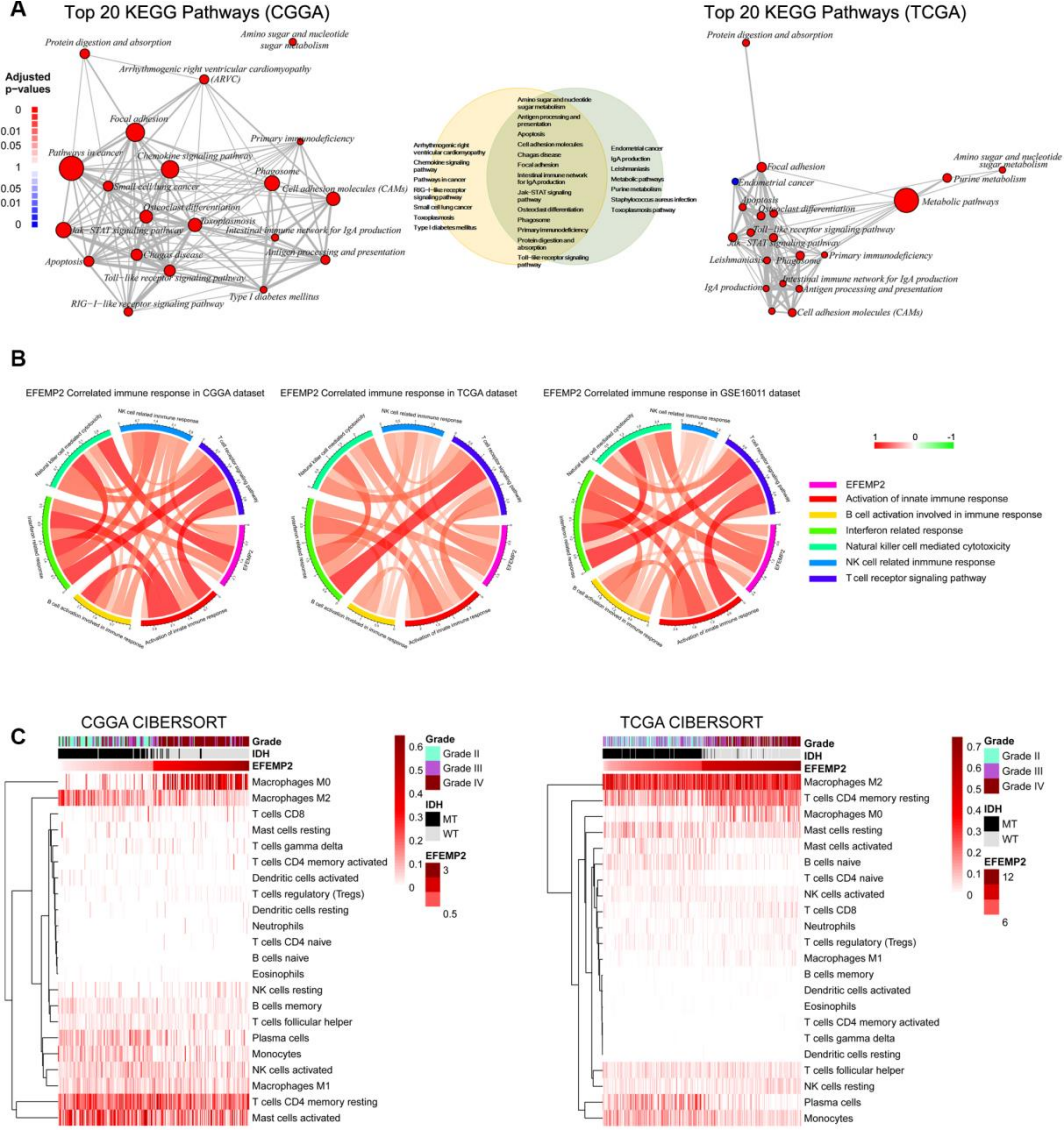
***EFEMP2* is closely correlated with immune microenvironment of glioma.** (**A**) Top 20 KEGG pathways derived from Gene Ontology analyses for *EFEMP2* in CGGA and TCGA dataset. Thirteen KEGG pathways are overlap between the top 20 pathways of each dataset. (**B**) Pearson correlation analysis of *EFEMP2* expression levels and immune responses in CGGA and TCGA datasets. Color depth and width of the bands represent the degrees of correlation. (**C**) Component types of the immune cells infiltrated into glioma are analyzed with CIBERSORT in CGGA and TCGA datasets.

All the above results confirmed the role of *EFEMP2* as an indicator of more malignant phenotype of glioma and worse outcomes of the patients. To annotate the detailed oncogenic biological processes contributed by *EFEMP2* or its protein product, we performed GO (Gene Ontology) analyses using the correlation (R value) between expression of *EFEMP2* and other transcriptome genes in both CGGA and TCGA datasets with *HTSanalyzeR* package developed by Wang, X. et al. [31] Comparison of the top 20 significantly enriched pathways in CGGA dataset and TCGA dataset revealed 13 overlapped terms, including three pathways directly correlated with immunological responses (Figure 5A). To further evaluate the influence of *EFEMP2* on tumor immunology, we performed Pearson correlation analysis and found that *EFEMP2* exhibited significant correlations with most of the critical immunology actions, especially interferon related response and natural killer cell mediated cytotoxicity. The distinct correlation between high expression of *EFEMP2* and immunology processes was consistent among CGGA, TCGA and GSE16011 datasets (Figure 5B).

Components of the immune cells infiltrated into tumors finally decide the immunological responses in which tumor cells are supported or attacked by the immune cells. We estimated the abundance of various types of immune cell with CIBERSORT for CGGA and TCGA cohort (Figure 5C). Unexpectedly, samples with higher *EFEMP2* expression exhibited apparent concordance with encirclement of macrophages in M0 phase instead of M2 phase. This result is, to some extent, in contrast to previous perceptions, which proposed that assembly of the tumor-supportive M2 macrophages was associated with more malignant properties of tumors [32, 33]. The canonical M1 versus M2 dichotomy was not found in samples with either the higher third or lower third *EFEMP2* expressions. Whereas, expression levels of *EFEMP2* showed a quite specific efficacy to distinguish M0-like macrophages (Figure 5C).

**Figure 6:**
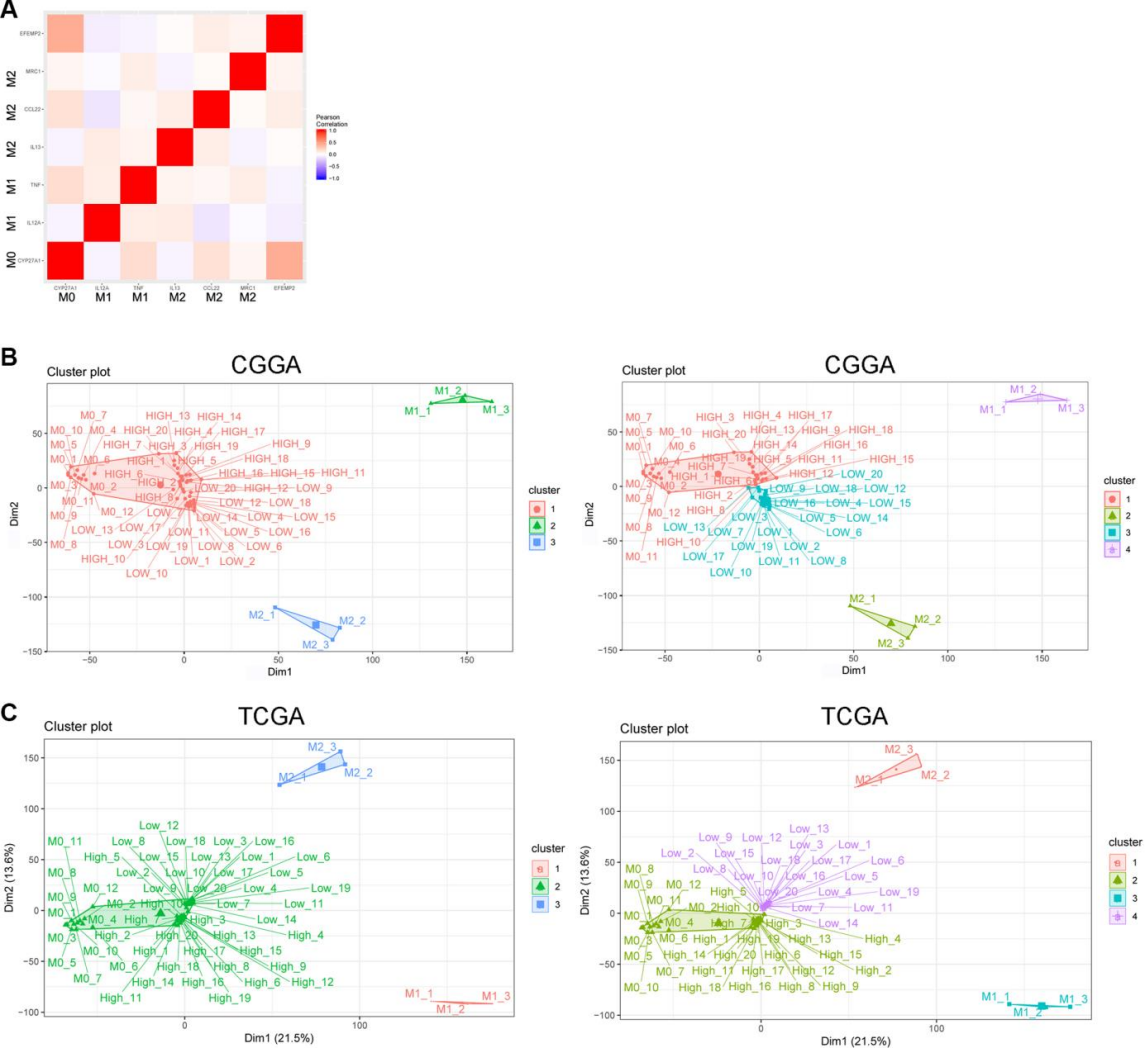
***EFEMP2* indicates assembly of M0 macrophage.** (**A**) Correlation analysis of *EFEMP2* and representative molecular of M0 (*CYP27A1*), M1 (*IL12A*, *TNF*) and M2 (*IL13*, *CCL22*, and *MRC1*) phenotype. (**B**) K-Means clustering (cluster = 3 or 4) based on whole genome expression profiling of M0, M1 and M2 phenotype from the dataset of a variety of resting and activated human immune cells (GSE22886) and CGGA RNA sequencing data. The samples named as “HIGH” were the top 20 samples with highest expression of *EFEMP2* in CGGA. The samples named as “LOW” were the top 20 samples with lowest expression of *EFEMP2* in CGGA. (**C**) K-Means clustering (cluster = 3 or 4) based on whole genome expression profiling of M0, M1 and M2 phenotype from the dataset of a variety of resting and activated human immune cells (GSE22886) and TCGA RNA sequencing data. The samples named as “HIGH” were the top 20 samples with highest expression of *EFEMP2* in TCGA. The samples named as “LOW” were the top 20 samples with lowest expression of *EFEMP2* in TCGA.

We chose representative markers of M0 (*CYP27A1*), M1 (*IL12A*, *TNF*) and M2 (*IL13*, *CCL22*, and *MRC1*) phenotype from several reports [34, 35] and found in CGGA RNA sequencing data that *EFEMP2* had stronger correlation with *CYP27A1* (M0) than with *IL12A*, *TNF* (M1), *IL13*, *CCL22*, or *MRC1* (M2) (Figure 6A).

Furthermore, we performed K-Means clustering based on whole genome expression profiling of M0, M1 and M2 phenotype from the dataset of a variety of resting and activated human immune cells (GSE22886) and CGGA glioma RNA sequencing data. When we set the cluster number as 3, all the CGGA samples (including the top 20 samples with highest expression of *EFEMP2* and the 20 samples with lowest expression of *EFEMP2*) gathered with M0 subtype and showed large differentiation with M1 or M2 subtype. When the cluster number was set as 4, the top 20 glioma samples with highest expression of *EFEMP2* specifically gathered with M0 phenotype (Figure 6B). These results suggested that glioma samples were more likely enriched of macrophages of M0 phenotype, and the samples with higher *EFEMP2* expression, which are the most malignant glioma (Figures 1, 2 showed the malignant phenotype of the samples with higher *EFEMP2* expression), were particularly prone to exhibit M0 features. The same preference was also observed in TCGA dataset (Figure 6C).

We analyzed the correlation of the expression levels of *EFEMP2* and immune checkpoints and did not find significant correlations between *EFEMP2* and typical immune checkpoints (Supplementary Figure 1).

## DISCUSSION

Although the M1 versus M2 dichotomy of TAM was widely recognized in numerous cancers and macrophages of M2 subtype were reported mainly gathered in high grade glioma, innovative and sound evidences supporting that abundant of nonpolarized M0 macrophages rather than M1 or M2 macrophages assembly in glioblastoma were proposed recently [17]. Other facts also suggested the complex nature of the glioma associated macrophages, and these cells might not fit well into the M1/M2 polarization dogma [16, 36, 37]. This is quite different from the traditional theory in other tumors that the alternatively activated or M2 type macrophages were pro-tumorigenic and contributed to the malignancy of tumor.

Here, we proposed a specific biomarker of assembly of M0 subtype. Definition a precise indicator of M0 macrophages will benefit the dissection of the immunological environment of glioma cells and resolving of the poor effects of immunotherapies in malignant glioma treatments.

Protein product of *EFEMP2* containing four EGF2 domains and six calcium-binding EGF2 domains is a member of fibulin family [38]. Fibulins are involved in various biological processes, such as embryonic development and organogenesis, hemostasis and thrombosis, fibrogenesis, tissue homeostasis, and remodeling [39]. Furthermore, fibulins may work as signaling transducer by directly or indirectly interacting with cellular membrane receptors and hence be a mediator of signaling pathways that regulating cell behaviors like cell morphology, growth, adhesion, and motility [40]. *EFEMP2* exhibits oncogenic activity such as promoting proliferation of lung cancer cells [19]. Expression level of this gene is closely correlated with outcomes of colorectal cancer [20]. Moreover, protein product of *EFEMP2* could exhibit its oncogenic activity in mutant p53-dependent or -independent manners [19]. *EFEMP2* has been proposed as a potential serum biomarker for the early detection of colorectal cancer [20]. Based on the pro-tumorigenic features of *EFEMP2* in other tumors and our analysis in glioma, we propose that *EFEMP2* might be used as a marker of the glioma subtype enriched of M0 macrophage.

Compared with CD14+ circulating blood cells of glioblastoma patients, blood coagulation function was more activated in glioblastoma-infiltrating CD14+ cells [41]. Furthermore, procoagulation factor fibrinogen-like protein 2 was demonstrated promoting expansion of M2 macrophages in glioblastoma [41]. These novel findings suggested that participants of blood coagulation might also play critical role in immunological regulation contributing to glioma initiation or progression. Protein product of *EFEMP2* was implicated in blood coagulation processes. The distinct correlation of *EFEMP2* expression level and assembly of M0 cells implied the role of *EFEMP2* in governing the fates of macrophages infiltrated into glioma. Recruiting and polarization of TAM were mainly mediated by chemoattraction, including chemokines, ligands of complement receptors, neuro-transmitters and ATP [9]. The specific localization of *EFEMP2* protein product in extracellular spaces supports EFEMP2 to act as a regulator of chemoattraction.

## MATERIALS AND METHODS

### Tissue specimens and cell culture

Human glioma cell lines U251, U373 and U87 were grown in Dulbecco’s Modified Eagle Medium (DMEM) (Gibco) supplemented with 10% fetal bovine serum (FBS; Gibco) and pen/strep. All of these cells were maintained at 37 °C in a humidified atmosphere containing 5% CO_2_.

GBM specimens were derived from patients with GBM in Beijing Tiantan Hospital. This study was approved by the Institutional Review Boards of Beijing Tiantan Hospital, and written informed consent was obtained from all patients.

### Immunohistochemistry (IHC)

For IHC staining, brain tumor sections were incubated with the EFEMP2 (1:250, Abcam, USA) antibody for 2 hour at room temperature after deparaffinization, rehydration, antigen retrieval, quenching of endogenous peroxidase and blocking. The images were captured with Axio Imager 2 (Zeiss) after 3,30-diaminobenzidine staining.

### Establishment of cell lines stably expressing or knocking down EFEMP2

The lentivirus constructing of overexpressing or knockdown EFEMP2 was obtained from GeneChem Co. Ltd. (Shanghai, PR, China). U251 cells were plated in 6 wells dishes at 20-30% confluence and infected with EFEMP2 overexpression lentivirus (termed as EFEMP2) or a negative control (termed as NC). EFEMP2 knockdown lentivirus (termed as sh-*EFEMP2*) or a scramble control (termed as sh-NC) was used to infect U87 cells, respectively. Pools of stable transductions were generated by selection using puromycin (2 μg/mL) for 2 weeks. *EFEMP2* expression was confirmed by RT-PCR, and the levels of EFEMP2 protein were measured by Western blotting.

### RNA extraction, reverse transcription-quantitative realtime PCR (RT-qPCR)

Total RNA was isolated using Trizol reagent (Invitrogen, USA). First-strand cDNA was generated using the RevertAid First Strand cDNA Synthesis Kit (Thermo scientific, USA). Real-time PCR was performed in the 7500 Fast Real-Time PCR System (Applied Biosystems, USA) using SYBR™ Select Master Mix (Applied Biosystems, Thermo scientific, USA), and the sequences of gene-specific primers were as follows: *EFEMP2*-sense, 5′-GAGTGTCTGACCATCCCTGAG-3′ and *EFEMP2*-antisense, 5′-GCCGTGTAGGTCGTTGATGAC-3′; GAPDH-sense, 5′-ATGGGGAAGGTGAAGGTCG-3′ and GAPDH-antisense, 5′-GGGGTCATTGATGGCAACAATA-3′. GAPDH was employed as an endogenous control for mRNA.

### Western blotting analysis

Total cellular proteins were lysed by RIPA lysis buffer (Beyotime, China). The protein extractions were harvested and quantified by bicinchoninic acid (BCA) analysis (Beyotime, China). Protein extractions were separated by 10% SDS-PAGE and transferred onto polyvinylidene fluoride (PVDF) membranes (Millipore, USA). After incubation with antibodies specific for EFEMP2 (1:1000, Abcam, USA) or GAPDH (1:1000, Proteintech, USA), the membranes were then incubated with peroxidase (HRP)-conjugated secondary antibody. After washes, bands were detected using the Chemi-DocTM XRS + (Bio-Rad, USA). GAPDH was used as a loading control.

For the conditioned medium, one 10 cm dish of 90% confluent cells was washed thoroughly with PBS twice and incubated for 48 h in 6 ml DMEM without serum. The conditioned medium from cells was collected and then centrifuged at 1000 rpm for 5 min to remove supernatant cells. Add 10% final concentration of trichloroacetic acid (TCA) to conditioned medium and incubate at 4 °C overnight. Centrifuge at 16,000g for 30 minutes to pellet protein. Remove supernatant and replace with 1 ml of cold acetone. Centrifuge at 16,000g for 15 minutes and allow pellet to dry in fume hood. Protein pellets were boiled with 100 μL of loading buffer at 95 °C for 10 min followed by Western blotting.

### The 5-ethynyl-2′-deoxyuridine incorporation assay

Based on the protocol outlined in the manual of the 5-ethynyl-2-deoxyuridine (EdU) labeling/detection kit (RiboBio, Guangzhou, PR, China), Cells of the control and experimental groups were digested and inoculated into a 96-well plate. After 24h of culture, labeling medium with 50 μM of EdU was added to the cell culture, and was incubated for 2 h at 37 °C with 5% CO_2_. The cells were then fixed with 4% paraformaldehyde (pH 7.4) for 30 min and incubated with glycine for 5 min. After being washed with PBS, cells were stained with anti-EdU working solution at room temperature for 30 min. They were then washed with 0.5% Triton X-100 in PBS, and incubated with DAPI at room temperature for 3 min. Cells were then observed using fluorescent microscopy.

### Cell growth assay

The growth ability of GBM cells was evaluated using the Electric Cell-substrate Impedance Sensing (ECIS) (Applied Biophysics, USA), which can detect and quantify morphology changes in the sub-nanometer to micrometer range in real time. For ECIS measurement, 5 × 10^3^ cells in 300 μL of DMEM complete medium were seeded in fibronectin-coated gold microelectrodes in ECIS cultureware (8W10E) and cultured at 37 °C with 5% CO_2_. Cellular impedance was measured continuously at a single frequency of 16000 Hz. The data was presented as normalized impedance versus time.

### Genetic and clinical information

All the mRNA expressions, genetic alterations and clinical information in this work were derived from public cancers/glioma datasets including TCGA, GSE16011 and REMBRANDT, or CGGA dataset established and managed by our team. This study was approved by the Institutional Review Boards of Beijing Tiantan Hospital, and written informed consent was obtained from all patients. All methods were performed in accordance with the relevant guidelines and regulations of the Institutional Review Boards. The establishment and management of our CGGA databank had been introduced in our previous publications [42, 43]. The expression profiles of M0, M1 and M2 subtype of macrophages were derived from the dataset of a variety of resting and activated human immune cells (GSE22886, downloaded from CIBERSORT website with a filename of “LM22 ref”). In this dataset, whole genome expression profile of 22 human immune subsets including M0 macrophages (samples names were “Monocyte-Day7-1” to “Monocyte-Day7-12”), M1 macrophages (samples names were “classical or M1 activated macrophages”) and M2 macrophages (samples names were “Alternative or M2 activated macrophage”) were provided.

Genetic alterations of TCGA were called by "MutSigCV" pipeline [44] while alterations of CGGA were extracted from RNA-sequencing data of CGGA.and called by our customized pipeline named "SAVI2", which was described in our previously published paper [45].

### Statistical analysis

Data are presented as the mean ± standard deviation (SD). Comparisons were determined using unpaired Student’s t test (*P < 0.05, **P < 0.01 and ***P < 0.001) as indicated in individuals. Kaplan-Meier analysis was performed by *survival* package in R language and inspection of survival difference between different groups was performed by log-rank test. *TimeROC* package compiled in R language, developed by Paul Blanche [46] was used to predict patients’ 3-year-survival and 5-year-survival. Forest plots was generated with *forestplot* package (https://CRAN.R-project.org/package=forestplot). *Oncoprint* plots was generated with *ComplexHeatmap* package developed by Gu, Z. et al. [23] Gene Ontology analysis was performed with HTSanalyzeR developed by Wang X. et al. [31] Circos plots indicating the relationship between *EFEMP2* expression and specific immune pathways were generated by circlize package developed by Gu Z. et al. [47] Heatmaps demonstrating relationship between *EFEMP2* and immune cell fractions was plotted by pheatmap package developed by Raivo Kolde (https://CRAN.R-project.org/package=pheatmap). A p value less than 0.05 was considered to be statistically significant. The K-Means analysis was performed using the built-in function “kmeans” in R language.

## Supplementary Material

Supplementary Figure 1
