# Supplementary material for: EFEMP2 indicates assembly of M0 macrophage and more malignant phenotypes of glioma
Source: Aging (Albany NY). 2020 May 12;12(9):8397–412. doi: 10.18632/aging.103147 (PMC7244085; doi:10.18632/aging.103147)
Supplement: Supplementary Figure 1 [file aging-12-103147-s001..pdf]

SUPPLEMENTARY FIGURE

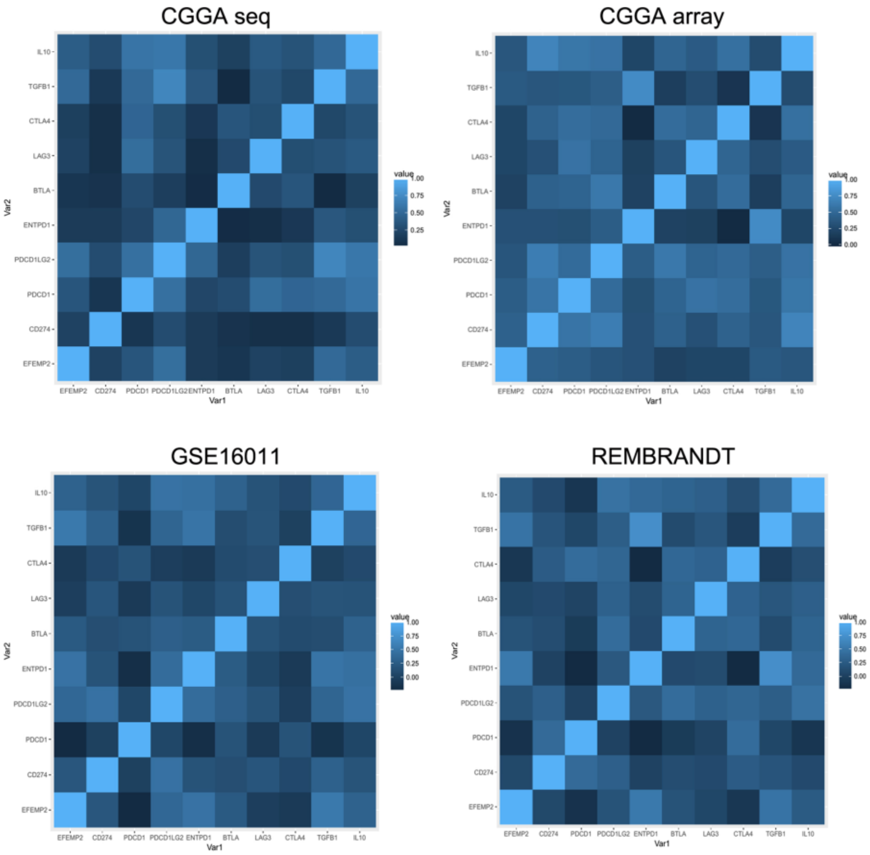

Supplementary Figure 1. Correlation of the expression levels of *EFEMP2* and immune checkpoints.
